# Diagnostic test accuracy for detecting *Schistosoma japonicum* and *S. mekongi* in humans: A systematic review and meta-analysis

**DOI:** 10.1371/journal.pntd.0009244

**Published:** 2021-03-17

**Authors:** Md. Obaidur Rahman, Miho Sassa, Natasha Parvin, Md. Rashedul Islam, Aya Yajima, Erika Ota

**Affiliations:** 1 Department of Global Health Nursing, Graduate School of Nursing Science, St. Luke’s International University, Tokyo, Japan; 2 Department of Global Health Policy, Graduate School of Medicine, The University of Tokyo, Tokyo, Japan; 3 Department of Parasitology, Institute of Tropical Medicine (NEKKEN), Nagasaki University, Nagasaki, Japan; 4 Department of Accounting and Information System, Hajee Mohammad Danesh Science & Technology University, Dinajpur, Bangladesh; 5 Division of Prevention, Center for Public Health Sciences, National Cancer Center, Tokyo, Japan; 6 Malaria and Neglected Tropical Diseases Unit, Division of Progammes of Disease Control, World Health Organization Western Pacific Regional Office, World Health Organization; PUCRS, BRAZIL

## Abstract

**Background:**

Most of national schistosomiasis elimination programmes in Asia are relying on stool examination, particularly Kato Katz stool examination technique for regular transmission monitoring. However, the Kato-Katz technique has shown low sensitivity for the detection of light-intensity infections, and therefore highly sensitive diagnostic tools are urgently required to monitor prevalence of infection in low transmission settings. The objective of this systematic review was to evaluate and synthesize the performance of diagnostic tests for detecting *Schistosoma japonicum* and *S*. *mekongi* infection in people living in endemic areas.

**Methodology/Principal findings:**

We comprehensively searched these nine electronic databases and other resources until July 2019, with no language or publication limits: PubMed, EMBASE, MEDLINE, Web of Science, BIOSIS Citation Index, HTA, CINAHL PLUS, The Cochrane Library, and PsycINFO. We included original studies that assessed diagnostic performance using antibody, antigen, and molecular tests with stool examination test as a reference standard. Two reviewers independently extracted a standard set of data and assessed study quality. We estimated the pooled estimates of sensitivity and specificity for each index test. We used diagnostic odds ratio to determine the overall accuracy and hierarchical summary receiver operating characteristics (HSROC) curve to assess the index tests performance.

Fifteen studies (*S*. *japonicum* [n = 13] and *S*. *mekongi* [n = 2]) testing 15,303 participants were included in the review. Five studies reported performance of enzyme-linked immunosorbent assay (ELISA), seven studies reported indirect hemagglutination assay (IHA), and four studies reported polymerase chain reaction (PCR) for detecting *S*. *japonicum*. The pooled sensitivity and specificity were 0.93 (95% CI: 0.84–0.98) and 0.40 (95% CI: 0.29–0.53) for ELISA, 0.97 (95% CI: 0.90–0.99) and 0.66 (95% CI: 0.58–0.73) for IHA, and 0.89 (95% CI: 0.71–0.96) and 0.49 (95% CI: 0.29–0.69) for PCR respectively. A global summary indicated the best performance for IHA, closely followed by ELISA. We were unable to perform meta-analysis for *S*. *mekongi* due to insufficient number of studies.

**Conclusions/Significance:**

IHA showed the highest detection accuracy for *S*. *japonicum*. Further studies are needed to determine the suitable diagnostic methods to verify the absence of transmission of *S*. *mekongi* and also to compare detection accuracy against more sensitive reference standards such as PCR.

## Author summary

Schistosomiasis remains a serious public health problem worldwide. Accurate diagnostic tests play a key role in control of schistosomiasis, especially in Asia where the prevalence and intensity of infection is low. As stool examination techniques, particularly the Kato-Katz technique has shown low sensitivity for the detection of light-intensity infections, highly sensitive diagnostics are urgently required to monitor prevalence of infection in low transmission settings. The purpose of this review was to assess and synthesize the performance of diagnostic tests for detecting schistosomiasis in people living in endemic areas in Asia. A comprehensive search, without any limit of language, date or types of publication, were performed for both- multiple electronic databases and other resources to identify the eligible studies. Robust analytical approaches such as diagnostic meta-analysis, HSROC curve, and diagnostic odds ratio, were used to provide more diagnostic accuracy of index tests. We assessed performance of three diagnostic tests (ELISA, IHA, and PCR) for detecting infection with *S*. *japonicum* using stool examination as a reference standard. In these meta-analyses, IHA showed the highest detection accuracy, followed by ELISA. We could not perform meta-analysis for *S*. *mekongi* due to insufficient number of studies.

## Introduction

Schistosomiasis is an acute and chronic parasitic disease caused by blood flukes of the genus *Schistosoma*. More than 200 million people, including 111 million school-aged children (SAC) and 95 million adults, are estimated to be at risk of infection globally [1]. People are infected when cercaria, the larval form of the parasite, that are released by the intermediate host freshwater snail penetrate the skin during contact with infested water [2]. Transmission occurs when infected people contaminate freshwater sources with their excreta containing parasite eggs, which hatch in water.

There are two major forms of schistosomiasis–intestinal and urogenital–caused by four main species of *Schistosoma*. *Schistosoma mansoni* (intestinal) and *S*. *haematobium* (urogenital) are endemic in Africa, South America, the Caribbean, and the Middle East. *S*. *japonicum* (intestinal) is currently endemic in China, Indonesia and the Philippines and was once endemic in Japan. *S*. *mekongi* (intestinal) is endemic in Cambodia and Lao People’s democratic Republic [3]. In Asia, more than 2.8 million people are estimated to be at risk and require preventive chemotherapy for schistosomiasis in 2017 [4].

Countries in Asia have a long history of efforts to control schistosomiasis. Japan eliminated schistosomiasis, with the last reported human case in 1977, mainly through intensive snail control and environmental modification [5]. China and the Philippines started national schistosomiasis control programmes in 1950 and 1960s respectively, focusing on snail control and health education then later on mass drug administration (MDA) once praziquantel was proven effective against schistosomes and it became widely available in 1990s [6,7]. Cambodia and Lao People’s Democratic Republic initiated annual MDA campaigns in the mid-1990s continuing to date [8]. As a result, prevalence and associated morbidity of the infection reduced significantly in many parts of endemic areas in Asia. Elimination of schistosomiasis in Asia is now considered within reach and all endemic countries are accelerating their efforts to interrupt transmission of schistosomiasis [5,6,9,10].

In general, intestinal schistosomiasis can be diagnosed by three different approaches: 1) parasitological methods to detect Schistosome eggs or miracidium in fecal samples; 2) immunological test to detect immunological responses to Schistosome antigens in specimens including serum, urine, feces, and saliva; and 3) pathological methods to measure schistosomiasis-associated morbidities by clinical and biomedical markers. Among them, the Kato Katz stool examination technique, which detects eggs in stool by staining a sieved fecal sample and examining it under a microscope remains the gold standard test for detection of intestinal schistosomiasis recommended by the World Health Organization [11]. However, it is widely recognized that Kato-Katz technique shows low sensitivity for the detection of light-intensity infections, and hence more sensitive diagnostic tools are urgently required to continue monitoring impacts of interventions in low prevalence settings and ultimately to verify the absence of transmission of schistosomiasis [12,13].

A number of new diagnostic tools have been developed for detection and diagnosis of schistosomiasis in recent years, some of which are commercially available. However, their performance has been largely validated only for *S*. *mansoni* and *S*. *haematobium* [14,15]. Usability of new serological tests in Asian settings requires careful validation as there are many co-endemic parasites, particularly trematodes, in schistosomiasis-endemic areas that might cause cross reaction. Systematic review and meta-analysis of diagnostic techniques for population-based monitoring and verification of interruption of transmission is currently ongoing but it only targets *S*. *mansoni* and *S*. *haematobium*. There are a few review studies on existing diagnostic tools for *S*. *japonicum* but it only concerns China and focuses on immunological tests especially ELISA and IHA [16,17]. A comprehensive systematic review and meta-analysis of data pertaining to all existing diagnostic tools for *S*. *japonicum* and *S*. *mekongi* was urgently needed to help determine the most appropriate diagnostic tools for population-level monitoring of intervention impacts in low-prevalence setting and ultimately to verify interruption of transmission of *S*. *japonicum* and *S*. *mekongi*.

### Review objective/questions

The key objective of this systematic review was to evaluate and synthesize the performance of diagnostic tests for detecting infection with *S*. *japonicum* and *S*. *mekongi* in people living in endemic areas of these diseases. More explicitly, we addressed the following review questions:

- which diagnostic tools/techniques are adequate to verify the absence of transmission of *S*. *japonicum* in humans? and
- which diagnostic tools/techniques are adequate to verify the absence of transmission of *S*. *mekongi* in humans?

## Methods

### Study registration

The protocol was registered in the international prospective register of systematic review (PROSPERO registration number- CRD42020162558). The guideline of Cochrane Handbook for Systematic Reviews of Diagnostic Test Accuracy and preferred reporting items for systematic review and meta-analysis of diagnostic test accuracy (PRISMA-DTA) statement were followed in this review [18,19].

### Study identification

To identify the relevant studies, we attempted to search both electronic databases and other resources. We executed a comprehensive search of the following nine electronic databases up to July 2019: PubMed, EMBASE, MEDLINE, Web of Science, BIOSIS Citation Index, Health Technology Assessment (HTA), CINAHL PLUS, The Cochrane Library, and PsycINFO. We used controlled vocabulary and text words for each database. We did not limit our search by language, date or types of publication in order not to miss any published studies. Moreover, we checked reference lists of all included studies and relevant systematic reviews to identify further studies. The details of the search strategies for each database are shown in S1 Appendix.

### Study selection

Two reviewers independently screened the titles and abstracts of all retrieved studies. Thereafter, they independently critically reviewed all potentially relevant studies at the full-text screening stage to assess the eligibility in detail. During both stages, any discrepancies were resolved through discussion or by a third reviewer. A study was included if the study met all of the following criteria:

1. Study participants were individuals residing in endemic countries of *S*. *japonicum* and *S*. *mekongi*, regardless of their age, gender or occupation
2. It assessed diagnostic performance of any of the index tests (antibody tests, antigen tests, and molecular tests) with a parasitological detection test such as stool examination and miracidium hatching test as a reference standard
3. It reported sensitivity and specificity or included data that enabled calculation of sensitivity and specificity
4. Types of study included were cross-sectional studies, case-control studies and cohort studies but not case-control studies with healthy control or qualitative studies. Commentaries, case studies, editorials, expert opinions, or symposium proceedings were also excluded.

The details of the eligibility criteria were reported in the study protocol elsewhere. We recorded the reasons of all studies excluded in the full-text screening stage and reported in a PRISMA flow diagram. We used EndNote software and Rayyan QRCI tool in the study selection process [20].

### Data extraction

Two reviewers independently extracted a standard set of data including study characteristics, participant characteristics, description and results of index tests and reference standards from each of the selected studies and was cross-checked. Similarly to the study selection process, disagreements were resolved through discussion or by a third reviewer. We reported key characteristics of the included studies in a separate table. The characteristic data included, but was not limited to: author, year of publication, study location, study setting, study design, number of participants, study year, age of participants, types of study population, prior treatment for schistosomiasis, index tests (diagnostic method, condition, species and cut-off value), reference standards (with details), examined disease, and reported outcomes. True positive, false positive, false negative, true negative, sensitivity and specificity of the index tests, and the prevalence of schistosome infection by the reference standards were considered as primary data (see S1 and S2 Tables).

### Quality assessment

The Quality Assessment of Diagnostic Accuracy Studies tool-2 (QUADAS-2) was used to assess the risk of bias and applicability of the studies. The QUADAS-2 consisted of the following four main domains (see S2 Appendix): patient selection, index test, reference standard, and flow and timing. We did not make tailoring QUADAS-2 tool as all signaling questions of the tool adequately cover the issues for the systematic review. Firstly, we piloted QUADAS-2 tool in a small number of studies and met the good agreement by two independent reviewers. Applying the tool, two independent reviewers assessed the quality of each study included in the review and any disagreements were resolved through discussion or by a third reviewer. A study was classified as a high-quality report if an original study did not have high risk of bias or high applicability concerns in any of the QUADAS-2 domains.

### Statistical analysis

We performed meta-analysis for all index tests if there were three or more studies with no substantial clinical heterogeneity among the studies. In the meta-analysis, we considered parasitological detection tests as reference standards because parasitological tests have been most widely used and it is known to deliver reliable results if they performed well. In order to estimate the sensitivity and specificity of each index tests, a reference standard was established by combining all stool examinations including Kato-Katz test, formol-ethyl acetate sedimentation concentration technique (FECT), and cellophane thick smear. We classified the data for each type of index tests by the reference standard. We estimated pooled estimates (with 95% confidence interval) of sensitivity, specificity, positive likelihood ratio, negative likelihood ratio, positive predictive value (PPV) and negative predictive value (NPV) for each index test using a bivariate model. We used diagnostic odds ratio (DOR) using the DerSimonian-Laird random effect model to determine the overall accuracy, when making comparisons between index tests. A hierarchical summary of receiver operating characteristic (HSROC) curve using HSROC model was employed to make comparisons of the diagnostic accuracy among different index tests. Moreover, forest plots and the 95% prediction regions in the HSROC curves were checked visually to assess the heterogeneity of the diagnostic accuracy between the included studies. To assess the reporting bias, we planned to use funnel plots if there were at least ten studies remaining. We used Review Manager 5.4 and Stata version 15.0/MP software to perform all statistical analyses.

## Results

### Study inclusion

**Fig 1:**
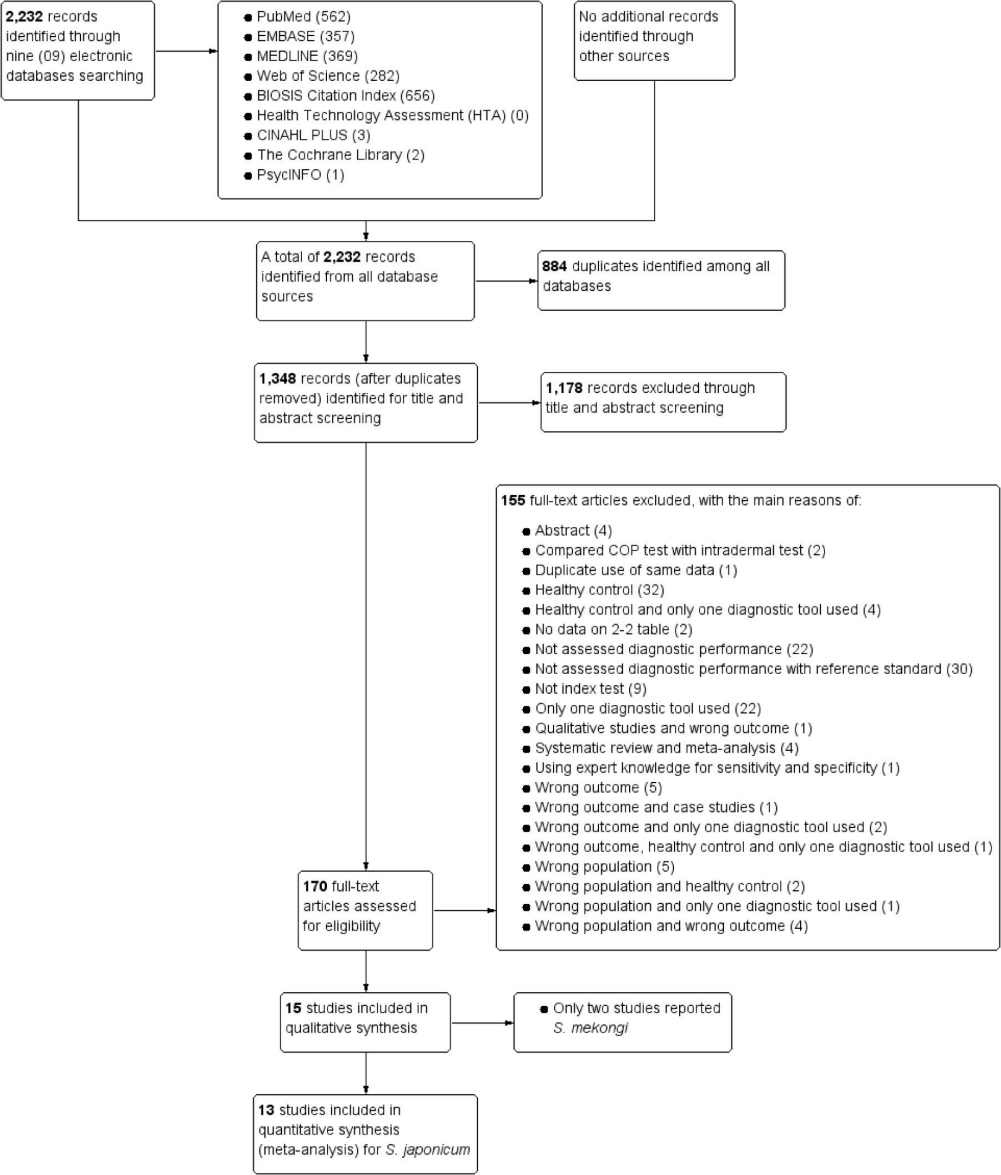
PRISMA flowchart of study selection.

We identified 2,232 potentially relevant articles from all targeted sources of databases. Of these, 884 articles were found to be duplicates. A total of 1,348 unique articles after excluding duplicates underwent title and abstract screening. Thereafter, 170 studies were assessed for eligibility in details. In full-text screening, 153 studies failed to meet our eligibility criteria and were excluded. The reasons for exclusion were reported in the PRISMA flowchart of study selection process (Fig 1). Finally, we found 15 eligible studies for narrative synthesis (Fig 1 and Tables 1 and 2). Of the 15 eligible studies, 13 reported *S*. *japonicum* and we performed a meta-analysis (Fig 1). The remaining 2 studies reported on *S*. *mekongi*. Due to limited data, we were unable to perform a meta-analysis for *S*. *mekongi* studies.

**Table 1:** Characteristics of studies included in meta-analysis for *S*. *japonicum*.

SL	Country (reference)	Study design	Study year	Total sample size	Participant’s age	Prior treatment with PZQ before	Index test	Reference standard
1	Philippines[23]	Cross-sectional study	2015	412	5–69 years		ELISA and ddPCR assay	Kato-Katz test (3 slides from single stool)
2	China[24]	Cross-sectional study	2010	142	6–68 years		PCR assay	Kato-Katz test (3 slides from single stool)
3	Philippines[25]	Cross-sectional study	2011	560	> = 4 years		qPCR assay	Kato-Katz test
4	China[26]	Cross-sectional study		2,732	5–76 years	Yes	DIGFA, DDIA, IHA-A, IHA-B	Stool examination
5	China[27]	Cross-sectional study		394	5–80 years	No	IHA, ELISA	Stool examination
6	Philippines[22]	Cross-sectional study	2015	452			ddPCR assay with stool, serum, urine, and saliva	Kato-Katz (6 slides from 2 stools) or Fecal ddPCR
7	China[28]	Cross-sectional study	2003	1,553	5–84 years		IHA, CDIFA	Kato-Katz (3 slides from single stool) and/or IHA
8	China[29]	Cross-sectional study	2013–2014	200	15–78 years		RPA, ELISA, IHA	Kato-Kato (3 slides from single stool)
9	China[30]	Cross-sectional study	2005	1864	6–65 years	No	DDIA, IHA-A, IHA-B	Stool examination
10	Philippine[31]	Cross-sectional study		338			COPT, ELISA	MFCT
11	Philippines[32]	Cross-sectional study	1979	598	>6 years student		ELISA	MFCT
12	China[33]	Cross-sectional study		1,914	5–75 years		IHA, ELISA	Kato-Katz (6 slides from 2 stools)
13	China[34]	Cross-sectional study	2001–2006	3,533	5–65 years		IHA	Kato-Katz (3 slides from single stool)

Note: CDIFA-Colloidal dye immunofiltration assay; COPT-Circumoval precipitin test; DDIA-Dipstick dye immunoassay; ddPCR-Droplet digital polymerase chain reaction; DIGFA-Dot immunogold filtration assay; ELISA-Enzyme-Linked immuno sorbent assay; IHA-Indirect haemagglutination assay; IHA-A-Indirect haemagglutination assay test A; IHA-B-Indirect haemagglutination assay test B; MFCT-Modified methiolate-formaldehyde concentration technique; PCR-Polymerase chain reaction; qPCR- Real-time polymerase chain reaction; RPA-Recombinase polymerase amplification.

**Table 2:** Characteristics of included studies for *S*. *mekongi*.

SL	Country (reference)	Study design	Study year	Total sample size	Participant’s age	Prior treatment with PZQ before	Index test	Reference standard
1	Laos[35]	Cross sectional study	2011–2012	234	2–77 years		ELISA	Kato-Katz
2	Laos[36]	Cross-sectional survey	2006	485	> = 6 months		FECT	Kato-Katz

Note: ELISA-Enzyme-Linked immuno sorbent assay; FECT-Formol-ethyl acetate sedimentation concentration technique.

### Characteristics of the included studies

All included studies were cross-sectional studies (Tables 1 and 2). Most of the studies were from China (8 studies), 5 studies from Philippines, and 2 studies from Laos. All studies except for two studies (one focused on fishermen and another focused-on student) targeted all age groups in endemic communities, ranging from 4 to 84 years of age for *S*. *japonicum* and 2 to 77 years of age for *S*. *mekongi*. A total of 14,692 participants were included in the studies for detecting *S*. *japonicum* and 611 participants for *S*. *mekongi*. Five studies reported performance of enzyme-linked immunosorbent assay (ELISA), 7 studies reported indirect hemagglutination assay (IHA), and 4 studies reported polymerase chain reaction (PCR) for detecting *S*. *japonicum*. The sensitivity of ELISA, IHA, and PCR ranged from 59% to 100%, 76% to 100%, and 40% to 98% respectively and the specificity varied from 11% to 69% for ELISA, 36% to 83% for IHA, and 11% to 91% for PCR.

### Overall diagnostic accuracy

In quantitative comparison, we merged weightedly the odds ratios of ELISA, IHA, and PCR. The diagnostic odds ratio (DOR) was 9.0 (95% CI: 4.0–20) for ELISA, 60 (95% CI: 15–242) for IHA, and 8.0 (95% CI: 4.0–16) for PCR (Table 3). The results indicated that IHA outperformed both ELISA and PCR techniques.

**Table 3:** Summary estimates of diagnostic accuracy for *S*. *japonicum* (stool examination as a reference standard).

	ELISA			IHA			PCR		
	Estimate	95% CI		Estimate	95% CI		Estimate	95% CI	
Sensitivity	0.93	0.84	0.98	0.97	0.90	0.99	0.89	0.71	0.96
Specificity	0.40	0.29	0.53	0.66	0.58	0.73	0.49	0.29	0.69
Positive Likelihood Ratio	1.6	1.3	1.8	2.8	2.2	3.6	1.7	1.3	2.4
Negative Likelihood Ratio	0.17	0.08	0.35	0.05	0.01	0.17	0.23	0.11	0.49
Diagnostic Odds Ratio	9.0	4.0	20	60	15	242	8.0	4.0	16
Positive Predictive Value	0.43	0.40	0.45	0.30	0.27	0.32	0.35	0.33	0.38
Negative Predictive Value	0.92	0.91	0.94	0.96	0.94	0.97	0.91	0.89	0.93

Note: CI-Confidence interval; ELISA-Enzyme-Linked immuno sorbent assay; IHA-Indirect haemagglutination assay; PCR-Polymerase chain reaction.

### Pooled results of sensitivity and specificity

**Fig 2:**
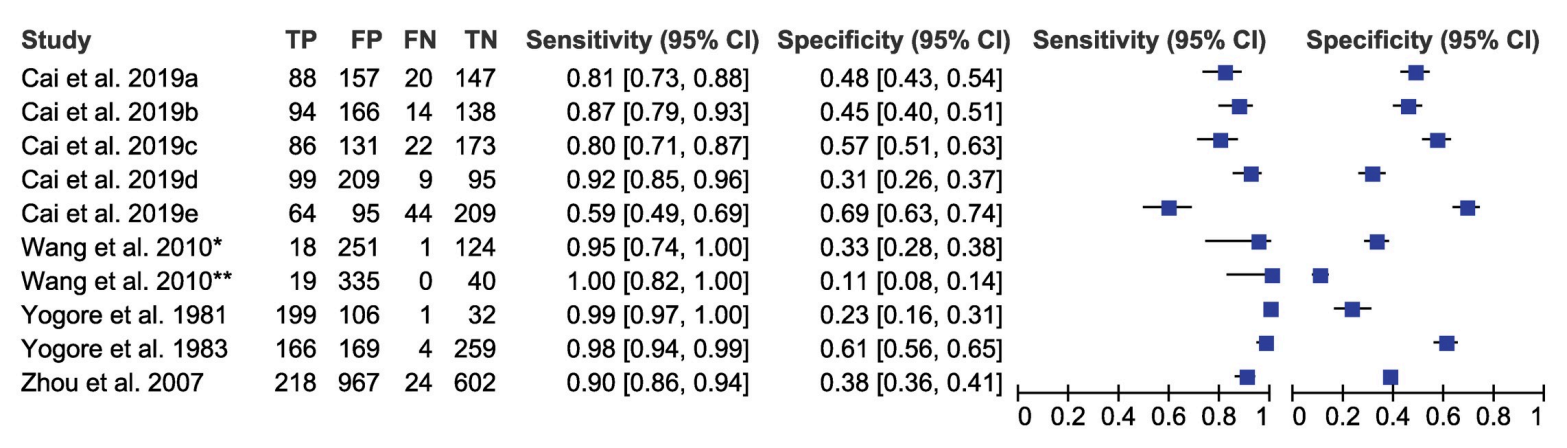
Forest plot of sensitivity/specificity of ELISA (*S*. *japonicum* with stool examination as a reference standard). Note: a-SjSAP5 + Sj23-LHD ELISA; b-SjSAP4 + Sj23-LHD ELISA; c-SjSAP5 ELISA; d-SjSAP4 ELISA; e-Sj23-LHD ELISA; *-AWA ELISA; **-rsj29 ELISA.

**Fig 3:**
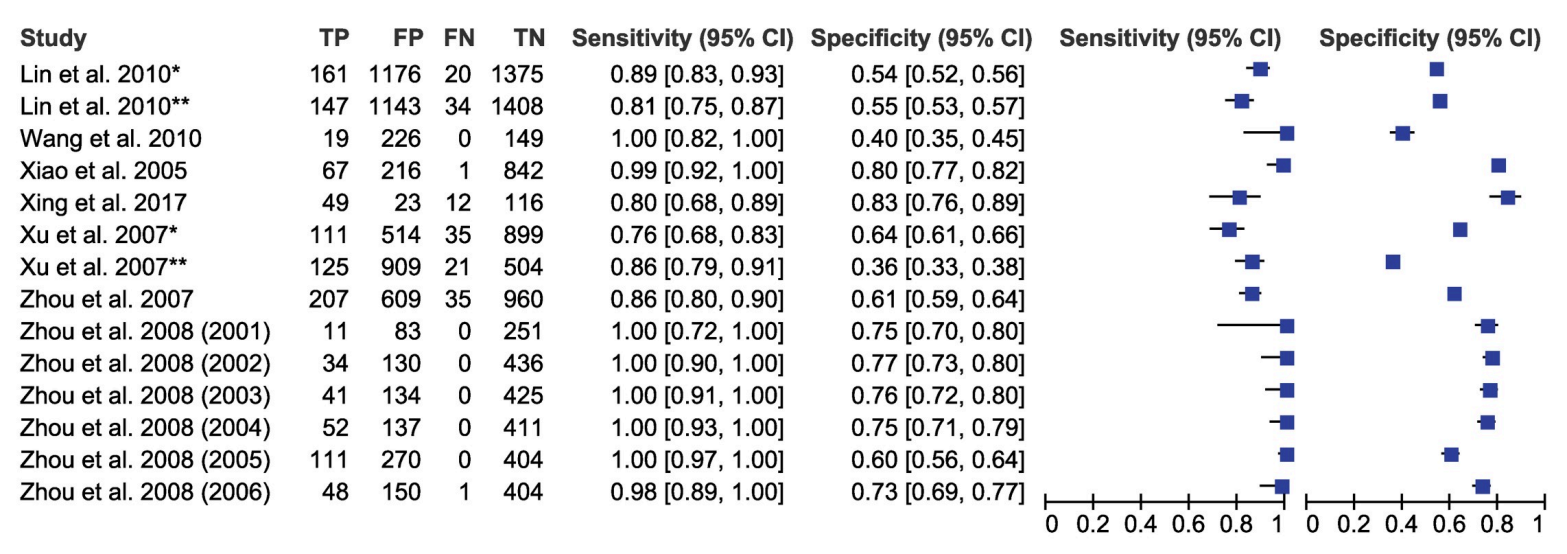
Forest plot of sensitivity/specificity of IHA (*S*. *japonicum* with stool examination as a reference standard). Note: *-IHA_A; **-IHA_B.

**Fig 4:**
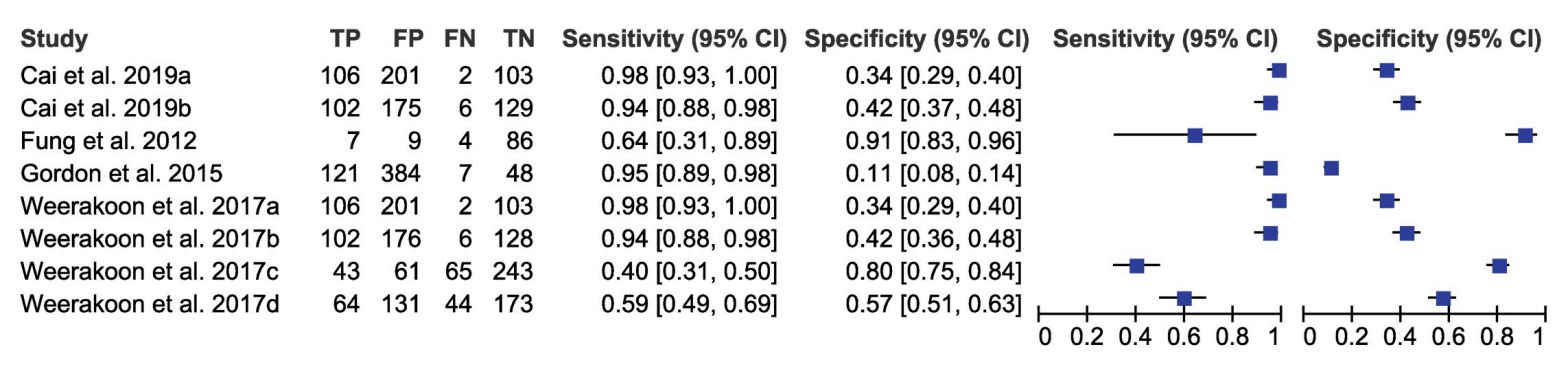
Forest plot of sensitivity/specificity of PCR (*S*. *japonicum* with stool examination as a reference standard). Note: a-Fecal ddPCR; b-Serum ddPCR; c-Urine ddPCR; d-Saliva ddPCR.

We pooled the sensitivity and specificity of ELISA, IHA, and PCR for detecting *S*. *japonicum* considering stool examination as reference standard. The sensitivity and specificity were 0.93 (95% CI: 0.84–0.98) and 0.40 (95% CI: 0.29–0.53) for ELISA, 0.97 (95% CI: 0.90–0.99) and 0.66 (95% CI: 0.58–0.73) for IHA, and 0.89 (95% CI: 0.71–0.96) and 0.49 (95% CI: 0.29–0.69) for PCR respectively (Figs 2–4 and Table 3). These results indicated that while all three diagnostic tools have high sensitivity above 0.89, they also have moderate specificity with IHA as the highest.

**Fig 5:**
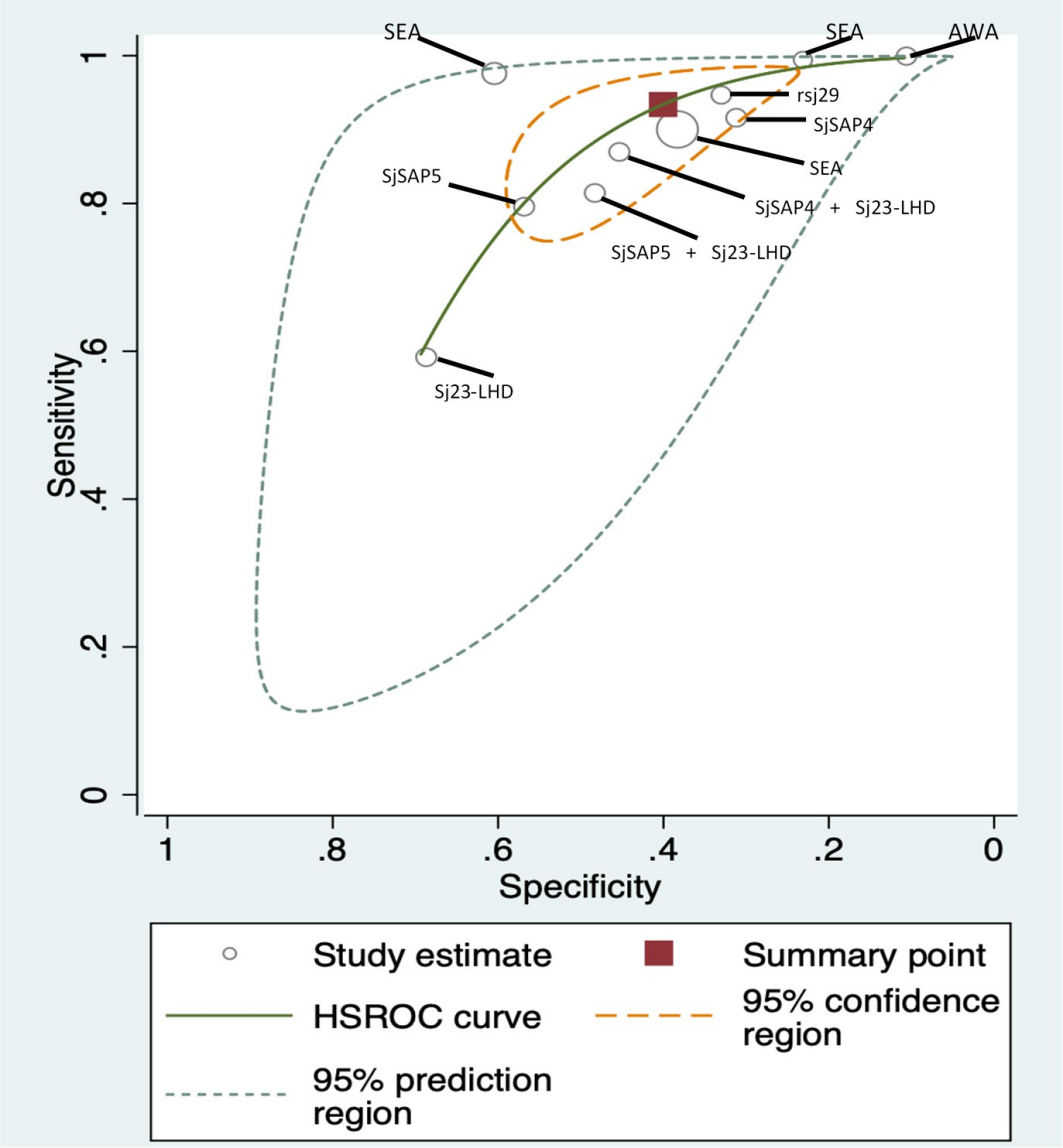
HSROC curve for ELISA (*S*. *japonicum* with stool examination as a reference standard).

**Fig 6:**
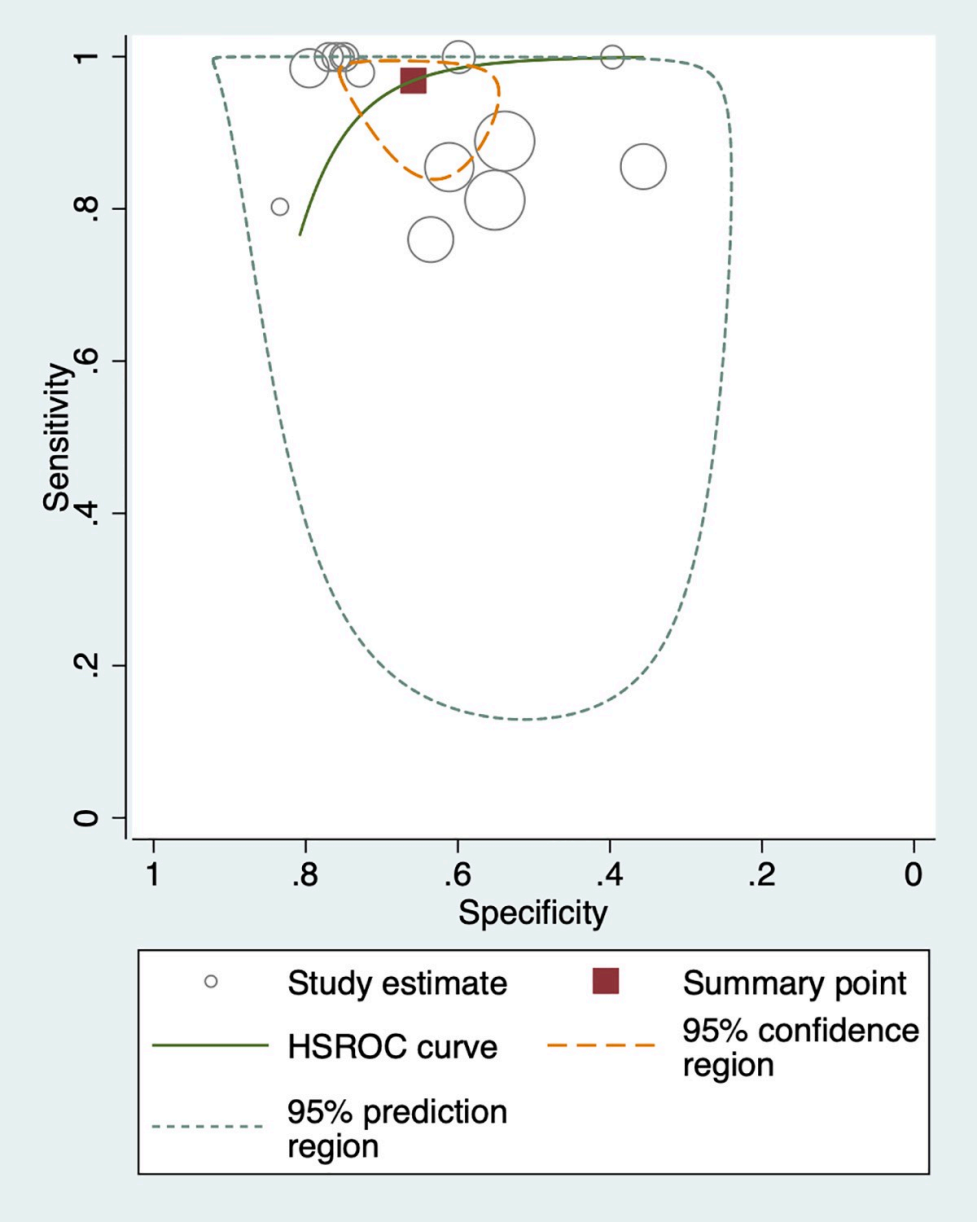
HSROC curve for IHA (*S*. *japonicum* with stool examination as a reference standard).

**Fig 7:**
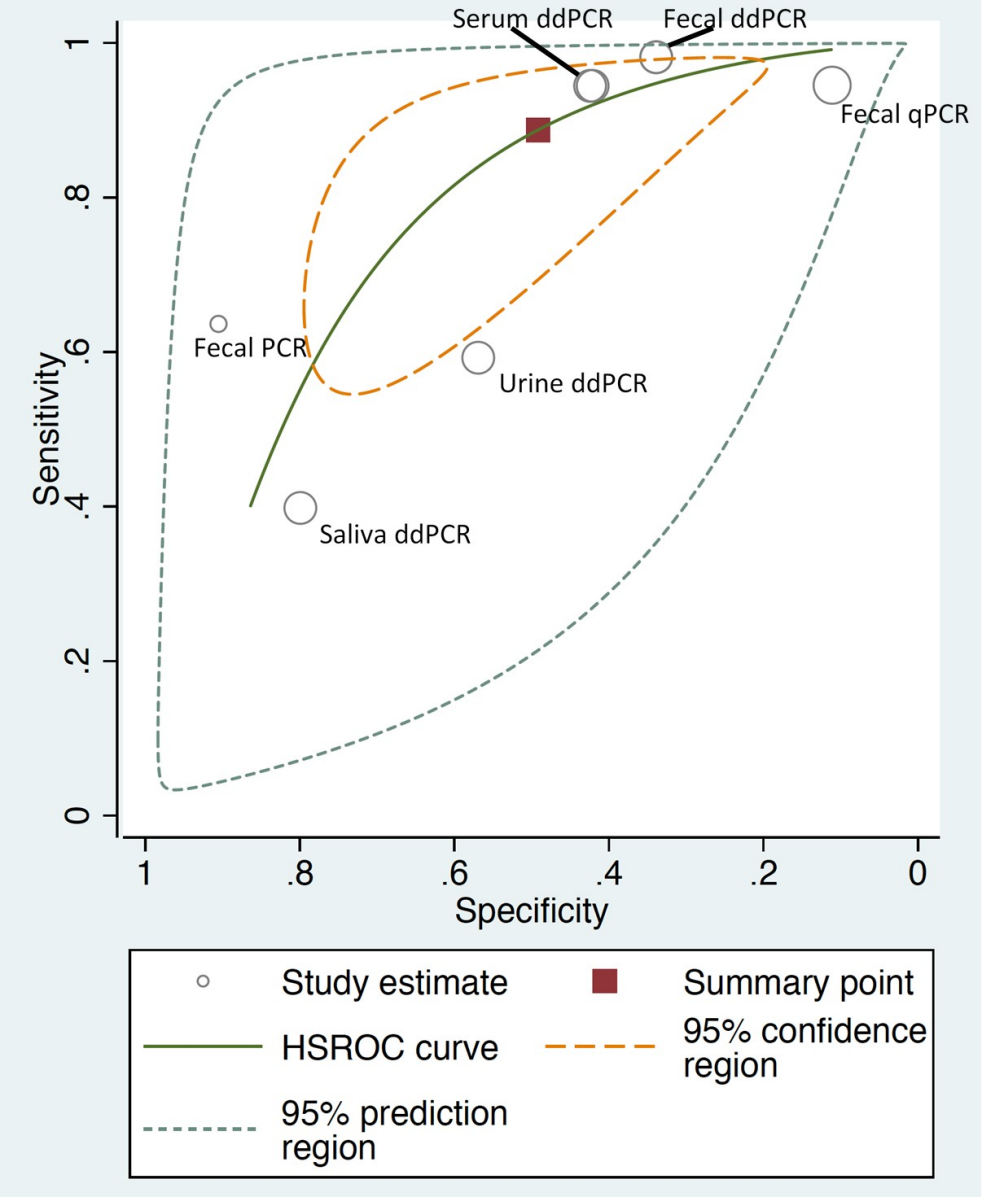
HSROC curve for PCR (*S*. *japonicum* with stool examination as a reference standard).

We used the HSROC curve that presented a global summary of the performance of diagnostic tools (Figs 5–7). IHA showed the best performance for diagnosis of *S*. *japonicum*, based on the highest summary sensitivity and specificity with the smallest 95% confidence region. This was followed by ELISA with similarly high sensitivity and small 95% confidence region, though slightly lower specificity. PCR too showed high sensitivity but low specificity and large 95% confidence region.

### Positive and negative likelihood ratios

We summarized positive likelihood ratio (PLR) and negative likelihood ratio (NLR) for ELISA, IHA, and PCR. IHA showed higher PLR (2.8 with 95% CI: 2.2–3.6) and lower NLR (0.05 with 95% CI: 0.01–0.17) compared to other techniques (Table 3).

### Positive and negative predictive values

We pooled PPV and NPV for ELISA, IHA, and PCR. The PPV and NPV were 0.43 (95% CI: 0.40–0.45) and 0.92 (95% CI: 0.91–0.94) for ELISA, 0.30 (95% CI: 0.27–0.32) and 0.96 (95% CI: 0.94–0.97) for IHA, and 0.35 (95% CI: 0.33–0.38) and 0.91 (95% CI: 0.89–0.93) for PCR respectively. These results indicated that IHA showed lower PPV and higher NPV compared to other techniques (Table 3).

### Quality assessment of bias and applicability

**Fig 8:**
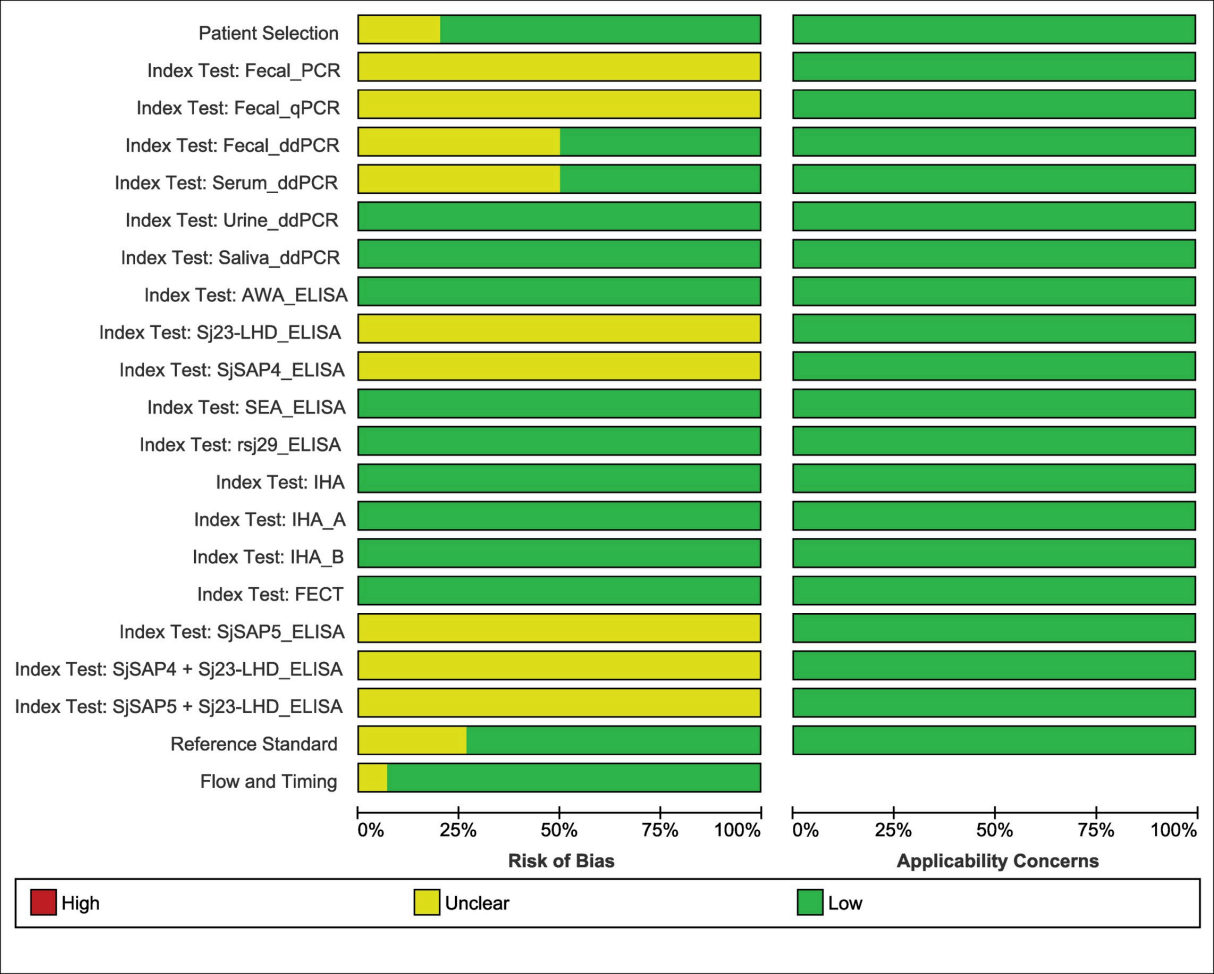
Risk of bias and applicability concerns graph: each domain presented as percentages across included studies.

**Fig 9:**
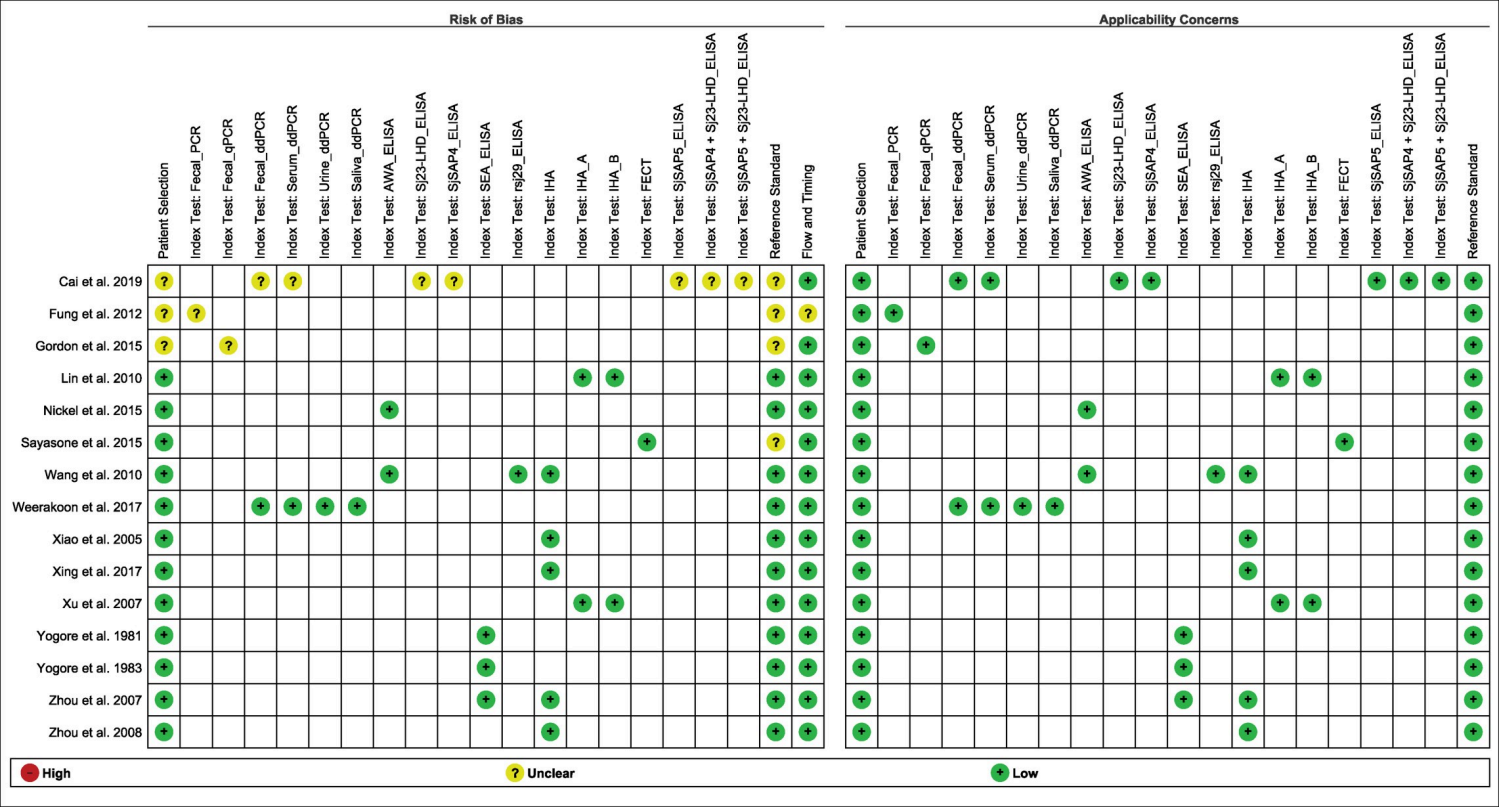
Risk of bias and applicability concerns summary for each domain of the included studies. Note: Blank cell represents the study not reported respective index test.

Based on QUADAS-2 assessment, no studies showed a high risk of bias or high applicability concern (Figs 8 and 9). Most of the studies had low risk of bias and all studies showed low applicability concern. There were very few studies with unclear risk of bias. However, most of the studies were classified as high-quality reports (Figs 8 and 9).

### Investigation of heterogeneity and publication bias

To assess the heterogeneity of the diagnostic accuracy between the included studies, we graphically assessed forest plots and the 95% prediction regions in the HSROC curves (Figs 2–7). These figures showed that higher heterogeneity remained among these studies. Though we planned to use funnel plots to assess reporting bias, we were unable to perform analysis due to insufficient number of studies for each index test.

## Discussion

### Summary of main results

This study is the first DTA study that comprehensively reviewed all available and relevant publications and assessed performance of diagnostic tests for detecting Asian schistosomiasis, namely infection with *S*. *japonicum* and *S*. *mekongi*, covering all endemic areas of these diseases. Stool examination techniques were used as the reference standard for meta-analysis. The total of 15 studies was found to meet our inclusion criteria, and data from these studies were used in this review.

Our results showed that the diagnostic performance of ELISA, IHA and PCR for detecting *S*. *japonicum* varied greatly by publications. The diagnostic accuracy based on the pooled estimates for *S*. *japonicum* with stool examinations as a reference standard showed that all three tools present high sensitivity above 0.89 with IHA the highest (sensitivity: 97%, 95% CI [90–99]) but moderate specificity with IHA the highest (specificity: 66%, 95% CI [58–73]).

The ELISA test for *S*. *japonicum* detected a large proportion of infections detected by stool examination (sensitivity 93%, 95% CI [84–98]). However, it also diagnosed a large proportion of those identified uninfected by stool examinations as positive (specificity 40%, 95% CI [29–53]). This might be due to a number of possibilities: first of all, stool examination, particularly Kato Katz technique, is known to show low sensitivity for the detection of light-intensity infections; secondly, ELISA included in this review detects antibody response to Schistosome antigens in specimens and therefore is unable to differentiate current and past infections, unlike stool examination; it only detects current infections. Thirdly, the studies included in the analysis applied seven different antigens for detecting antibodies response and the sensitivity of each antigen differs [21]. Among studies using ELISA, the one using soluble egg antigen (SEA) demonstrated the highest sensitivity (90–100%) though specificity varied (23–61%) (Fig 5). Similarly, low performance of PCR in both pooled sensitivity (89%, 95% CI [71–96]) and pooled specificity (49%, 95% CI [29–69]) against the stool examination might be explained by the variations of test accuracy caused by the type of samples such as stool, serum and saliva.

Our meta-analysis found that the two immunological assays, namely IHA and ELISA, have similarly high accuracy for the detection of *S*. *japonicum* compared to stool examination. This is consistence with two other meta-analyses that assessed the accuracy of ELISA and IHA in reference to parasitological examination for *S*. *japonicum* in China [16,17]. However, all the studies using IHA that were included in our study were performed in China, and therefore there is a possibility that the results may not be generalizable to other settings.

As the number of studies was limited, we were unable to generate summary estimates for the circumoval precipitin test (COPT), colloidal dye immunofiltration assay (CDIFA), dipstick dye immunoassay (DDIA), dot immunogold filtration assay (DIGFA), dipstick with latex immunochromatographic assay (DLIA), and recombinase polymerase amplification (RPA). In addition, we were unable to conduct a meta-analysis for *S*. *mekongi* due to insufficient number of studies. However, among those studies available for *S*. *mekongi*, the circulating anodic antigen (CAA) test for urine sample showed high performance with its sensitivity (84%) and specificity (100%) when compared to the reference standard of combined results of Kato-Katz, urine CAA and serum CAA.

### Strengths and limitations of the review

We have reviewed and evaluated the performance of existing diagnostic tests for schistosomiasis in Asia, namely *S*. *japonicum* and *S*. *mekongi*, for which systematic review and meta-analysis is limited or non-existent. We did not limit our search by publication year or language. We followed PRISMA-DTA review methodology. Two reviewers independently performed data extraction to eliminate bias.

The absence of highly sensitive diagnostic tools for detection of schistosomiasis in low transmission settings has been a major challenge in Asia where different ways of stool examination techniques are used as reference standards to validate diagnostic accuracy of various tools. Our study encountered a challenge due to a variety of slides and/or stools used in the reference standard test among the studies. The number of studies to be included in the review was dramatically reduced when we selected studies that used stool examination as a reference standard. Besides, even within stool examination, a variety of methods were used as a reference standard, such as Kato-Katz, FECT, MFCT, stool examination, stool direct smear, and cellophane thick smear. Sensitivity of stool examinations is also known to differ by the number of slides and stool samples examined per individual sample. All these factors undoubtedly contributed to a large variety of diagnostic efficacies with a wide range of estimated performance found in this review.

Another potential cause of a wide range of diagnostic performance in this review was the pooling of immunological studies using ELISA with different antigens to detect *S*. *japonicum* infection due to a limited number of studies available for each type of antigen. We also pooled studies using different types of PCR (ddPCR, qPCR and conventional PCR) with several types of specimens (stool, serum, urine and saliva). The diagnostic performance of PCR for detecting Schistosoma infection is known to differ by the sample type [21,22], which may also have had an effect on our results.

### Application of the meta-analysis to a review question

All schistosomiasis-endemic countries in Asia are accelerating efforts to interrupt transmission of the disease with 2030 as a regional target for disease elimination, through annual mass drug administration campaigns, eliminating open defecation and improving hygiene, snail control and veterinary interventions. In the absence of a gold standard to monitor prevalence of infections in low transmission settings, most of the national schistosomiasis elimination programmes in Asia are regularly monitoring impacts of interventions using Kato Katz technique. However, in order to document success and eventually verify achievement of interruption of transmission of Asian schistosomiasis, namely *S*. *japonicum* and *S*. *mekongi*, more sensitive and specific diagnostic methods are urgently needed.

All the studies included in our review showed low applicability concern based on the assessment using QUADAS-2. Studies conducted in low prevalence settings (i.e., 53.3% of studies were conducted in areas with below 20% prevalence of infection) were included in the review, therefore our results can be applied in the low transmission settings as well. After screening of the studies using selection criteria, only studies using IHA, ELISA or PCR with stool examination as a reference standard for detection of *S*. *japonicum* infection were included in our review. Therefore, our results cannot be applied to other diagnostic techniques or detection of Schistosome species other than *S*. *japonicum*. Moreover, poor and/or lack of reporting of participant characteristics, particularly their intensity of infection and status of praziquantel treatment, limited our risk of bias assessment.

Most of endemic countries in Asia have limited parasitic laboratory capacity. When determining the appropriate diagnostic tools for surveillance of *S*. *japonicum* and *S*. *mekongi* for verification of interruption of transmission, therefore, it is important to consider not only sensitivity and specificity of each tool but also existing laboratory capacity and financial needs to upgrade laboratory capacity to adopt new tools.

## Conclusion

Our DTA study to review all available and relevant publications and assess performance of diagnostic tests for detecting infection with *S*. *japonicum* and *S*. *mekongi* was challenged by a limited number of published studies that meet selection criteria. Among the three tests evaluated for *S*. *japonicum* infection using stool examination as a reference standard, IHA showed the highest detection accuracy, closely followed by ELISA, in moderate and low transmission settings in Asia. We were unable to conduct a meta-analysis for *S*. *mekongi* due to insufficient number of studies.

Researchers of epidemiological studies on Asian schistosomiasis, particularly *S*. *mekongi*, in the future should be encouraged to use the STARD guidelines for planning, performing and reporting their studies. This will help systematic reviewers to better synthesis the data and enable more comprehensive conclusions on risk of bias in studies. Further studies to enable comparison of accuracy of index tests against more sensitive reference standards such as PCR are also needed to determine the suitable diagnostic methods to more accurately verify the absence of transmission of schistosomiasis.

## Supporting information

S1 PRISMA Checklist PRISMA-DTA for abstract checklist. (DOC)

S2 PRISMA Checklist PRISMA-DTA checklist. (DOC)

S1 Appendix Search strategy of electronic databases. (DOCX)

S2 Appendix Quality Assessment of Diagnostic Accuracy Studies tool-2 (QUADAS-2). (DOCX)

S1 Table Index test specific 2x2 data (TP, FP, FN, TN) of studies reported *S*. *japonicum*. (DOCX)

S2 Table Index test specific 2x2 data (TP, FP, FN, TN) of studies reported *S*. *mekongi*. (DOCX)

## References

[1] WHO. Schistosomaisis Countries x indicators [cited 9 July 2019]. Available from: https://www.who.int/neglected_diseases/preventive_chemotherapy/sch/db/?units=minimal&region=all&country=ken&countries=ken&year=20172017.

[2] Colley DG, Bustinduy AL, Secor WE, King CH. Human Schistosomiasis. Lancet. 2014;383(9936):2253–64. 10.1016/S0140-6736(13)61949-2 24698483 PMC4672382

[3] WHO. Schistosomiasis [cited 9 July 2019]. Available from: https://www.who.int/en/news-room/fact-sheets/detail/schistosomiasis2019.

[4] WHO. Schistosomiasis, Countries x indicators [cited 9 July 2019]. Available from: https://www.who.int/neglected_diseases/preventive_chemotherapy/sch/db/?units=minimal&region=WPR&country=all&countries=all&year=2018.2018.

[5] Tanaka H, Tsuji M. From discovery to eradication of Schistosomiasis in Japan: 1847–1996. Int J Parasitol. 1997;27(12):1465–80. 10.1016/s0020-7519(97)00183-5 9467732

[6] Olveda DU, Li Y, Olveda RM, Lam AK, McManus DP, Chau TN, et al. Bilharzia in the Philippines: past, present. and future Int J Infect Dis. 2014;18:52–6. 10.1016/j.ijid.2013.09.011 24211228

[7] Sun LP, Wang W, Hong Q-B, Li S-Z, Liang Y-S, Yang H-T, et al. Approaches being used in the national Schistosomiasis elimination programme in China: a review. Infect Dis Poverty. 2017;6(1):55. 10.1186/s40249-017-0271-9 28292327 PMC5351197

[8] WHO. Expert Consultation to Accelerate Elimination of Asian Schistosomiasis, Shanghai, China, 22–23 May 2017: meeting report. Manila: WHO Regional Office for the Western Pacific; 2017.

[9] Gordon CA, Kurscheid J, Williams GM, Clements ACA, Li Y, Zhou X-N, et al. Asian Schistosomiasis: Current Status and Prospects for Control Leading to Elimination. Trop Med Infect Dis. 2019;4(1). 10.3390/tropicalmed4010040 PMC6473711 30813615

[10] Khieu V, Sayasone S, Muth S, Kirinoki M, Laymanivong S, Ohmae H, et al. Elimination of Schistosomiasis Mekongi from Endemic Areas in Cambodia and the Lao People’s Democratic Republic: Current Status and Plans. Trop Med. Infect Dis. 2019;4(1). 10.3390/tropicalmed4010030 30736431 PMC6473609

[11] WHO. Schistosomiasis: progress report 2001–2011 and strategic plan 2012–2020. Geneva, Switzerland 2013.

[12] Bärenbold O, Raso G, Coulibaly JT, N’Goran EK, Utzinger J, Vounatsou P. Estimating sensitivity of the Kato-Katz technique for the diagnosis of Schistosoma mansoni and hookworm in relation to infection intensity. PLoS Negl Trop Dis. 2017;11(10):e0005953–e. 10.1371/journal.pntd.0005953 28976979 PMC5643140

[13] King CH. It’s time to dispel the myth of "asymptomatic" schistosomiasis. PLoS Negl Trop Dis. 2015;9(2):e0003504–e. 10.1371/journal.pntd.0003504 25695740 PMC4335065

[14] Ashton RA, Stewart BT, Petty N, Lado M, Finn T, Brooker S, et al. Accuracy of circulating cathodic antigen tests for rapid mapping of Schistosoma mansoni and S. haematobium infections in Southern Sudan Trop Med Int Health. 2011;16(9):1099–103. 10.1111/j.1365-3156.2011.02815.x 21692957

[15] Rubaba O, Chimbari MJ, Soko W, Manyangadze T, Mukaratirwa S. Validation of a urine circulating cathodic antigen cassette test for detection of Schistosoma haematobiumin uMkhanyakude district of South Africa. Acta Trop. 2018;182:161–5. 10.1016/j.actatropica.2018.02.029 29486172

[16] Wang W, Li Y, Li H, Xing Y, Qu G, Dai J, et al. Immunodiagnostic efficacy of detection of Schistosoma japonicum human infections in China: a meta analysis. Asian Pac J Trop Med. 2012;5(1):15–23. 10.1016/S1995-7645(11)60238-1 22182637

[17] Zhu H, Yu C, Xia X, Dong G, Tang J, Fang L, et al. Assessing the diagnostic accuracy of immunodiagnostic techniques in the diagnosis of Schistosomiasis japonica: a meta-analysis. Parasitol Res. 2010;107(5):1067–73. 10.1007/s00436-010-1970-3 20607287

[18] Macaskill P, Gatsonis C, Deeks J, Harbord R, Takwoingi Y. Cochrane handbook for systematic reviews of diagnostic test accuracy. Version 09 0 London: The Cochrane Collaboration. 2010.

[19] McInnes MD, Moher D, Thombs BD, McGrath TA, Bossuyt PM, Clifford T, et al. Preferred reporting items for a systematic review and meta-analysis of diagnostic test accuracy studies: the PRISMA-DTA statement. JAMA. 2018;319(4):388–96. 10.1001/jama.2017.19163 29362800

[20] Ouzzani M, Hammady H, Fedorowicz Z, Elmagarmid A. Rayyan—a web and mobile app for systematic reviews. Sys Rev. 2016;5(1):210. 10.1186/s13643-016-0384-4 27919275 PMC5139140

[21] Cai P, Weerakoon KG, Mu Y, Olveda RM, Ross AG, Olveda DU, et al. Comparison of Kato Katz, antibody-based ELISA and droplet digital PCR diagnosis of Schistosomiasis japonica: Lessons learnt from a setting of low infection intensity. PLoS Negl Trop Dis. 2019;13(3):e0007228–e. 10.1371/journal.pntd.0007228 30830925 PMC6417743

[22] Weerakoon KG, Gordon CA, Williams GM, Cai P, Gobert GN, Olveda RM, et al. Droplet Digital PCR Diagnosis of Human Schistosomiasis: Parasite Cell-Free DNA Detection in Diverse Clinical Samples. J Infect Dis. 2017;216(12):1611–22. 10.1093/infdis/jix521 29029307

[23] Cai P, Weerakoon KG, Mu Y, Olveda RM, Ross AG, Olveda DU, et al. Comparison of Kato Katz, antibody-based ELISA and droplet digital PCR diagnosis of schistosomiasis japonica: Lessons learnt from a setting of low infection intensity. PLoS Negl Trop Dis. 2019;13(3):e0007228. 10.1371/journal.pntd.0007228 30830925 PMC6417743

[24] Fung MS, Xiao N, Wang S, Carlton EJ. Field evaluation of a PCR test for Schistosoma japonicum egg detection in low-prevalence regions of China. Am J Tropl Med Hyg. 2012;87(6):1053–8. 10.4269/ajtmh.2012.12-0177 23109374 PMC3516074

[25] Gordon CA, Acosta LP, Gobert GN, Olveda RM, Ross AG, Williams GM, et al. Real-time PCR demonstrates high prevalence of Schistosoma japonicum in the Philippines: implications for surveillance and control. PLoS Negl Trop Dis. 2015;9(1):e0003483. 10.1371/journal.pntd.0003483 25606851 PMC4301913

[26] Lin DD, Xu J, Liu HY, Zeng XJ, Liu YM, Xie SY, et al. Comparative evaluation of five test kits for antibody detection in Schistosoma japonicum endemic areas of Poyang Lake region. [Zhongguo ji sheng chong xue yu ji sheng chong bing za zhi]. Chin J Parasit Parasit Dis. 2010;28(6):439–43. 21500532

[27] Wang P, Ren CP, Wang TP, Shen JJ. Evaluation of recombinant 29,000 extra membranous protein for the immunodiagnosis of Schistosomiasis japonica. [Zhongguo ji sheng chong xue yu ji sheng chong bing za zhi]. Chin J Parasit Parasit Dis. 2010;28(4):284–6. 21137315

[28] Xiao X, Wang T, Ye H, Qiang G, Wei H, Tian Z. Field evaluation of a rapid, visually-read colloidal dye immunofiltration assay for Schistosoma japonicum for screening in areas of low transmission. Bull World Health Organ. 2005;83(7):526–33. 16175827 PMC2626292

[29] Xing W, Yu X, Feng J, Sun K, Fu W, Wang Y, et al. Field evaluation of a recombinase polymerase amplification assay for the diagnosis of Schistosoma japonicum infection in Hunan province of China. BMC Infect Dis. 2017;17(1):6. 10.1186/s12879-016-2128-4 28222680 PMC5320755

[30] Xu J, Chen NG, Feng T, Wang EM, Wu XH, Chen HG, et al. Effectiveness of routinely used assays for the diagnosis of Schistosomiasis japonica in the field. [Zhongguo ji sheng chong xue yu ji sheng chong bing za zhi]. Chin J Parasit Parasit Dis. 2007;25(3):175–9. 18038771

[31] Yogore MG Jr, Lewert RM, Blas BL. Schistosomiasis japonica in Barrio San Antonio, Basey, Samar, in the Philippines. V. The enzyme-linked immunosorbent assay(ELISA) compared with quantitative stool examination and the circumoval precipitin(COP) test. Am J Trop Med Hyg. 1981;30(6):1252–62. 10.4269/ajtmh.1981.30.1252 6275726

[32] Yogore MG Jr, Lewert RM, Blas BL. Sero epidemiology of schistosomiasis japonica by elisa enzyme linked immuno sorbent assay in the philippines 1. Underestimation by stool examination of the prevalence of infection in school children. Am J TropMed Hyg. 1983;32(6):1322–34. 10.4269/ajtmh.1983.32.1322 6359910

[33] Zhou YB, Yang MX, Wang QZ, Zhao GM, Wei JG, Peng WX, et al. Field comparison of immunodiagnostic and parasitological techniques for the detection of Schistosomiasis japonica in the People’s Republic of China. Am J Trop Med Hyg. 2007;76(6):1138–43. 17556625

[34] Zhou YB, Yang MX, Tao P, Jiang QL, Zhao GM, Wei JG, et al. A longitudinal study of comparison of the Kato-Katz technique and indirect hemagglutination assay(IHA) for the detection of Schistosomiasis japonica in China, 2001–2006. Acta Trop. 2008;107(3):251–4. 10.1016/j.actatropica.2008.06.009 18675244

[35] Nickel B, Sayasone S, Vonghachack Y, Odermatt P, Marti H. Schistosoma mansoni antigen detects Schistosoma mekongi infection. Acta Trop. 2015;141:310–4. 10.1016/j.actatropica.2014.08.001 25116398

[36] Sayasone S, Utzinger J, Akkhavong K, Odermatt P. Repeated stool sampling and use of multiple techniques enhance the sensitivity of helminth diagnosis: a cross-sectional survey in southern Lao People’s Democratic Republic. Acta Trop. 2015;141:315–21. 10.1016/j.actatropica.2014.09.004 25225157

