# Supplementary material for: Diagnostic test accuracy for detecting Schistosoma japonicum and S. mekongi in humans: A systematic review and meta-analysis
Source: PLoS Negl Trop Dis. 2021 Mar 17;15(3):e0009244. doi: 10.1371/journal.pntd.0009244 (PMC7968889; doi:10.1371/journal.pntd.0009244)
Supplement: S1 Appendix — (DOCX) [file pntd.0009244.s003.docx]

**S1 Appendix. Search strategy of electronic databases.**

Search strategy of PubMed:

| **Search** | **Query** | **# items found** |
| --- | --- | --- |
| #1 | schistosoma [MeSH] OR schistosomiasis [MeSH] OR schistosom* [tw] OR “schistoma infection” [tw] OR “schistosom* infection” [tw] | 33,907 |
| #2 | bilharzia* [tw] OR bilharzio* [tw] OR “snail fever” [tw] OR “katayama fever” [tw] | 2,823 |
| #3 | “schistosoma japonicum” [MeSH] OR “schistosoma japonicum” [tw] OR “s japonicum” [tw] OR “S. japonicum” [tw] OR “schistosoma mekongi” [tw] OR “s mekongi” [tw] OR “S. mekongi” [tw] | 4,193 |
| #4 | (#1 OR #2) AND #3 | 4,163 |
| #5 | serology [MeSH] OR serology [tw] OR “serologic* test*” [tw] | 196,999 |
| #6 | “antigen* test*” [tw] OR ((antigens [MeSH] OR antigen [tw]) AND tests [tw]) | 121,733 |
| #7 | microscopy [MeSH] OR microscopy [tw] | 787,603 |
| #8 | “molecular test*” [tw] OR “laboratory test*” [tw] OR “rapid diagnostic test*” [tw] | 9,247 |
| #9 | ((antibody [MeSH] OR antibody [tw]) AND test* [tw]) OR “antibody test*” [tw] | 188,256 |
| #10 | #5 OR #6 OR #7 OR #8 OR #9 | 1,195,538 |
| #11 | #4 AND #10 | 561 |

Search strategy of EMBASE:

| **Search** | **Query** | **# items found** |
| --- | --- | --- |
| #1 | schistosoma:ti,ab,kw OR schistosomiasis:ti,ab,kw OR schistosom*:ti,ab,kw OR 'schistoma infection':ti,ab,kw OR 'schistosom* infection':ti,ab,kw | 32,683 |
| #2 | bilharzia*:ti,ab,kw OR bilharzio*:ti,ab,kw OR 'snail fever':ti,ab,kw OR 'katayama fever':ti,ab,kw | 3,075 |
| #3 | 'schistosoma japonicum':ti,ab,kw OR 's japonicum':ti,ab,kw OR 'S. japonicum':ti,ab,kw OR 'schistosoma mekongi':ti,ab,kw OR 's mekongi':ti,ab,kw OR 'S. mekongi':ti,ab,kw | 4,108 |
| #4 | (#1 OR #2) AND #3 | 4,060 |
| #5 | serology:ti,ab,kw OR 'serologic* test*':ti,ab,kw | 56,010 |
| #6 | 'antigen* test*':ti,ab,kw OR ((antigens:ti,ab,kw OR antigen:ti,ab,kw) AND tests:ti,ab,kw) | 38,632 |
| #7 | microscopy:ti,ab,kw | 502,016 |
| #8 | 'molecular test*':ti,ab,kw OR 'laboratory test*':ti,ab,kw OR 'rapid diagnostic test*':ti,ab,kw | 77,152 |
| #9 | antibody:ti,ab,kw AND test*:ti,ab,kw OR 'antibody test*':ti,ab,kw | 147,434 |
| #10 | #5 OR #6 OR #7 OR #8 OR #9 | 784,927 |
| #11 | #4 AND #10 | 357 |

Search strategy of MEDLINE:

| **Search** | **Query** | **# items found** |
| --- | --- | --- |
| S1 | TI (schistosoma OR schistosomiasis OR schistosom* OR “schistoma infection” OR “schistosom* infection” OR bilharzia* OR bilharzio* OR “snail fever” OR “katayama fever”) OR AB (schistosoma OR schistosomiasis OR schistosom* OR “schistoma infection” OR “schistosom* infection” OR bilharzia* OR bilharzio* OR “snail fever” OR “katayama fever”) | 30,921 |
| S2 | TI (“schistosoma japonicum” OR “s japonicum” OR “S. japonicum” OR “schistosoma mekongi” OR “s mekongi” OR “S. mekongi”) OR AB (“schistosoma japonicum” OR “s japonicum” OR “S. japonicum” OR “schistosoma mekongi” OR “s mekongi” OR “S. mekongi”) | 3,734 |
| S3 | S1 AND S2 | 3,696 |
| S4 | TI (serology OR “serologic* test*”) OR AB (serology OR “serologic* test*”) | 37,066 |
| S5 | TI (“antigen* test*” OR ((antigens OR antigen) AND tests)) OR AB (“antigen* test*” OR ((antigens OR antigen) AND tests)) | 61,449 |
| S6 | TI (microscopy) OR AB (microscopy) | 427,557 |
| S7 | TI (“molecular test*” OR “laboratory test*” OR “rapid diagnostic test*”) OR AB (“molecular test*” OR “laboratory test*” OR “rapid diagnostic test*”) | 47,655 |
| S8 | TI ((antibody AND test*) OR “antibody test*”) OR AB ((antibody AND test*) OR “antibody test*”) | 163,298 |
| S9 | S4 OR S5 OR S6 OR S7 OR S8 | 687,601 |
| S10 | S3 AND S9 | 369 |

Search strategy of Web of Science:

| **Search** | **Query** | **# items found** |
| --- | --- | --- |
| 1 | TI=(schistosoma OR schistosomiasis OR schistosom* OR “schistoma infection” OR “schistosom* infection” OR bilharzia* OR bilharzio* OR “snail fever” OR “katayama fever”) OR TS=(schistosoma OR schistosomiasis OR schistosom* OR “schistoma infection” OR “schistosom* infection” OR bilharzia* OR bilharzio* OR “snail fever” OR “katayama fever”) | 24,601 |
| 2 | TI=(“schistosoma japonicum” OR “s japonicum” OR “S. japonicum” OR “schistosoma mekongi” OR “s mekongi” OR “S. mekongi”) OR TS=(“schistosoma japonicum” OR “s japonicum” OR “S. japonicum” OR “schistosoma mekongi” OR “s mekongi” OR “S. mekongi”) | 2,764 |
| 3 | #1 AND #2 | 2,711 |
| 4 | TI=(serology OR “serologic* test*”) OR TS=(serology OR “serologic* test*”) | 30,232 |
| 5 | TI=(“antigen* test*” OR ((antigens OR antigen) AND tests)) OR TS=(“antigen* test*” OR ((antigens OR antigen) AND tests)) | 81,845 |
| 6 | TI=(microscopy) OR TS=(microscopy) | 933,874 |
| 7 | TI=(“molecular test*” OR “laboratory test*” OR “rapid diagnostic test*”) OR TS=(“molecular test*” OR “laboratory test*” OR “rapid diagnostic test*”) | 51,741 |
| 8 | TI=((antibody AND test*) OR “antibody test*”) OR TS=((antibody AND test*) OR “antibody test*”) | 137,955 |
| 9 | #4 OR #5 OR #6 OR #7 OR #8 | 1,174,348 |
| 10 | #3 AND #9 | 282 |

Search strategy of BIOSIS Citation Index:

| **Search** | **Query** | **# items found** |
| --- | --- | --- |
| 1 | TI=(schistosoma OR schistosomiasis OR schistosom* OR “schistoma infection” OR “schistosom* infection” OR bilharzia* OR bilharzio* OR “snail fever” OR “katayama fever”) OR TS=(schistosoma OR schistosomiasis OR schistosom* OR “schistoma infection” OR “schistosom* infection” OR bilharzia* OR bilharzio* OR “snail fever” OR “katayama fever”) | 27,673 |
| 2 | TI=(“schistosoma japonicum” OR “s japonicum” OR “S. japonicum” OR “schistosoma mekongi” OR “s mekongi” OR “S. mekongi”) OR TS=(“schistosoma japonicum” OR “s japonicum” OR “S. japonicum” OR “schistosoma mekongi” OR “s mekongi” OR “S. mekongi”) | 4,215 |
| 3 | #1 AND #2 | 4,088 |
| 4 | TI=(serology OR “serologic* test*”) OR TS=(serology OR “serologic* test*”) | 74,004 |
| 5 | TI=(“antigen* test*” OR ((antigens OR antigen) AND tests)) OR TS=(“antigen* test*” OR ((antigens OR antigen) AND tests)) | 96,403 |
| 6 | TI=(microscopy) OR TS=(microscopy) | 1,156,934 |
| 7 | TI=(“molecular test*” OR “laboratory test*” OR “rapid diagnostic test*”) OR TS=(“molecular test*” OR “laboratory test*” OR “rapid diagnostic test*”) | 28,957 |
| 8 | TI=((antibody AND test*) OR “antibody test*”) OR TS=((antibody AND test*) OR “antibody test*”) | 148,367 |
| 9 | #4 OR #5 OR #6 OR #7 OR #8 | 1,412,086 |
| 10 | #3 AND #9 | 656 |
