# Supplementary material for: Diagnostic test accuracy for detecting Schistosoma japonicum and S. mekongi in humans: A systematic review and meta-analysis
Source: PLoS Negl Trop Dis. 2021 Mar 17;15(3):e0009244. doi: 10.1371/journal.pntd.0009244 (PMC7968889; doi:10.1371/journal.pntd.0009244)
Supplement: S2 Appendix — (DOCX) [file pntd.0009244.s004.docx]

**S2 Appendix. Quality Assessment of Diagnostic Accuracy Studies tool-2 (QUADAS-2).**

**QUADAS-2: Risk of bias and applicability judgments**

DOMAIN 1: PATIENT SELECTION

A. Risk of Bias

- Was a consecutive or random sample of patients enrolled? (Yes/No/Unclear)
- Was a case-control design avoided? (Yes/No/Unclear)
- Did the study avoid inappropriate exclusions? (Yes/No/Unclear)
- Could the selection of patients have introduced bias? RISK: LOW/HIGH/UNCLEAR

B. Concerns regarding applicability

- Is there concern that the included patients do not match the review question? CONCERN: LOW/HIGH/UNCLEAR

DOMAIN 2: INDEX TEST(S)

If more than one index test was used, please complete for each test.

A. Risk of Bias

- Were the index test results interpreted without knowledge of the results of the reference standard? Yes/No/Unclear
- If a threshold was used, was it pre-specified? Yes/No/Unclear
- Could the conduct or interpretation of the index test have introduced bias? RISK: LOW /HIGH/UNCLEAR

B. Concerns regarding applicability

- Is there concern that the index test, its conduct, or interpretation differ from the review question? CONCERN: LOW /HIGH/UNCLEAR

DOMAIN 3: REFERENCE STANDARD

A. Risk of Bias

- Is the reference standard likely to correctly classify the target condition? Yes/No/Unclear
- Were the reference standard results interpreted without knowledge of the results of the index test? Yes/No/Unclear
- Could the reference standard, its conduct, or its interpretation have introduced bias? RISK: LOW /HIGH/UNCLEAR

B. Concerns regarding applicability

- Is there concern that the target condition as defined by the reference standard does not match the review question? CONCERN: LOW /HIGH/UNCLEAR

DOMAIN 4: FLOW AND TIMING

A. Risk of Bias

- Was there an appropriate interval between index test(s) and reference standard? Yes/No/Unclear
- Did all patients receive a reference standard? Yes/No/Unclear
- Did patients receive the same reference standard? Yes/No/Unclear
- Were all patients included in the analysis? Yes/No/Unclear
- Could the patient flow have introduced bias? RISK: LOW /HIGH/UNCLEAR
