# Supplementary material for: Diagnostic test accuracy for detecting Schistosoma japonicum and S. mekongi in humans: A systematic review and meta-analysis
Source: PLoS Negl Trop Dis. 2021 Mar 17;15(3):e0009244. doi: 10.1371/journal.pntd.0009244 (PMC7968889; doi:10.1371/journal.pntd.0009244)
Supplement: S1 Table — (DOCX) [file pntd.0009244.s005.docx]

**S1 Table. Index test specific 2x2 data (TP, FP, FN, TN) of studies reported *S. japonicum*.**

| **SL** | **Author (reference)** | **Reference standard** | **Index test** | **TP** | **FP** | **FN** | **TN** |
| --- | --- | --- | --- | --- | --- | --- | --- |
| 1 | Cai et al., 2019 [1] | Kato-Katz test (3 slides from single stool) | SjSAP5 + Sj23-LHD-ELISA | 88 | 157 | 20 | 147 |
|  |  |  | SjSAP4 + Sj23-LHD-ELISA | 94 | 166 | 14 | 138 |
|  |  |  | SjSAP5-ELISA | 86 | 131 | 22 | 173 |
|  |  |  | SjSAP4-ELISA | 99 | 209 | 9 | 95 |
|  |  |  | Sj23-LHD-ELISA | 64 | 95 | 44 | 209 |
|  |  |  | Fecal ddPCR | 106 | 201 | 2 | 103 |
|  |  |  | Serum ddPCR | 102 | 175 | 6 | 129 |
| 2 | Fung et al., 2012 [2] | Kato-Katz test (3 slides from single stool) | Fecal PCR | 7 | 9 | 4 | 86 |
| 3 | Gordon et al., 2015 [3] | Kato-Katz test | Fecal qPCR | 121 | 384 | 7 | 48 |
| 4 | Lin et al., 2010 [4] | Stool examination | IHA-A | 147 | 1143 | 34 | 1408 |
|  |  |  | IHA-B | 161 | 1176 | 20 | 1375 |
| 5 | Wang et al., 2010 [5] | Stool examination | rsj29-ELISA | 18 | 251 | 1 | 124 |
|  |  |  | AWA-ELISA | 19 | 335 | 0 | 40 |
|  |  |  | IHA | 19 | 226 | 0 | 149 |
| 6 | Weerakoon et al., 2017 [6] | Kato-Katz test (6 slides from 2 stools) | Fecal ddPCR | 106 | 201 | 2 | 103 |
|  |  |  | Serum ddPCR | 102 | 176 | 6 | 128 |
|  |  |  | Saliva ddPCR | 43 | 61 | 65 | 243 |
|  |  |  | Urine ddPCR | 64 | 131 | 44 | 173 |
| 7 | Xiao et al., 2005 [7] | Kato-Katz test (3 slides from single stool) | IHA | 67 | 216 | 1 | 842 |
| 8 | Xing et al., 2017 [8] | Kato-Kato test (3 slides from single stool) | IHA | 49 | 23 | 12 | 116 |
| 9 | Xu et al., 2007 [9] | Stool examination | IHA-A | 125 | 909 | 21 | 504 |
|  |  |  | IHA-B | 111 | 514 | 35 | 899 |
| 10 | Yogore et al., 1981 [10] | MFCT (quantitative stool examination) | ELISA | 199 | 106 | 1 | 32 |
| 11 | Yogore et al., 1983 [11] | MFCT | IgG-ELISA | 166 | 169 | 4 | 259 |
| 12 | Zhou et al., 2007 [12] | Kato-Katz test (6 slides from 2 stools) | SEA-ELISA | 218 | 967 | 24 | 602 |
|  |  |  | IHA | 207 | 609 | 35 | 960 |
| 13 | Zhou et al., 2008 [13] | Kato-Katz test (3 slides from single stool) | IHA (study conducted in 2001) | 11 | 83 | 0 | 251 |
|  |  |  | IHA (study conducted in 2002) | 34 | 130 | 0 | 436 |
|  |  |  | IHA (study conducted in 2003) | 41 | 134 | 0 | 425 |
|  |  |  | IHA (study conducted in 2004) | 52 | 137 | 0 | 411 |
|  |  |  | IHA (study conducted in 2005) | 111 | 270 | 0 | 404 |
|  |  |  | IHA (study conducted in 2006) | 48 | 150 | 1 | 404 |

*Note: ddPCR-Droplet digital polymerase chain reaction; ELISA-Enzyme-Linked immuno sorbent assay; IHA-Indirect haemagglutination assay; IHA-A-Indirect haemagglutination assay test A; IHA-B-Indirect haemagglutination assay test B; MFCT-Modified methiolate-formaldehyde concentration technique; PCR-Polymerase chain reaction; qPCR- Real-time polymerase chain reaction; SEA- Soluble egg raw antigen; TP-Ture positive; FP-False positive; FN-False negative; TN-True negative.*

**References**

1. Cai P, Weerakoon KG, Mu Y, Olveda RM, Ross AG, Olveda DU, et al. Comparison of Kato Katz, antibody-based ELISA and droplet digital PCR diagnosis of Schistosomiasis japonica: Lessons learnt from a setting of low infection intensity. PLoS Negl Trop Dis. 2019;13(3):e0007228.

2. Fung MS, Xiao N, Wang S, Carlton EJ. Field evaluation of a PCR test for Schistosoma japonicum egg detection in low-prevalence regions of China. Am J Trop Med Hyg. 2012;87(6):1053-8.

3. Gordon CA, Acosta LP, Gobert GN, Olveda RM, Ross AG, Williams GM, et al. Real-time PCR demonstrates high prevalence of Schistosoma japonicum in the Philippines: implications for surveillance and control. PLoS Negl Trop Dis. 2015;9(1):e0003483.

4. Lin DD, Xu J, Liu HY, Zeng XJ, Liu YM, Xie SY, et al. Comparative evaluation of five test kits for antibody detection in Schistosoma japonicum endemic areas of Poyang Lake region. [Zhongguo ji sheng chong xue yu ji sheng chong bing za zhi]. Chin J Parasit & Parasit Dis. 2010;28(6):439-43.

5. Wang P, Ren CP, Wang TP, Shen JJ. Evaluation of recombinant 29,000 extra membranous protein for the immunodiagnosis of schistosomiasis japonica. [Zhongguo ji sheng chong xue yu ji sheng chong bing za zhi]. Chin J Parasit & Parasit Dis. 2010;28(4):284-6.

6. Weerakoon KG, Gordon CA, Williams GM, Cai P, Gobert GN, Olveda RM, et al. Droplet Digital PCR Diagnosis of Human Schistosomiasis: Parasite Cell-Free DNA Detection in Diverse Clinical Samples. J Infect Dis. 2017;216(12):1611-22.

7. Xiao X, Wang T, Ye H, Qiang G, Wei H, Tian Z. Field evaluation of a rapid, visually-read colloidal dye immunofiltration assay for Schistosoma japonicum for screening in areas of low transmission. Bull World Health Organ. 2005;83(7):526-33.

8. Xing W, Yu X, Feng J, Sun K, Fu W, Wang Y, et al. Field evaluation of a recombinase polymerase amplification assay for the diagnosis of Schistosoma japonicum infection in Hunan province of China. BMC Infect Dis. 2017;17(1):6.

9. Xu J, Chen NG, Feng T, Wang EM, Wu XH, Chen HG, et al. Effectiveness of routinely used assays for the diagnosis of schistosomiasis japonica in the field. [Zhongguo ji sheng chong xue yu ji sheng chong bing za zhi]. Chin J Parasit & Parasit Dis. 2007;25(3):175-9.

10. Yogore MG, Jr., Lewert RM, Blas BL. Schistosomiasis japonica in Barrio San Antonio, Basey, Samar, in the Philippines. V. The enzyme-linked immunosorbent assay (ELISA) compared with quantitative stool examination and the circumoval precipitin (COP) test. Am J Trop Med Hyg. 1981;30(6):1252-62.

11. Yogore MG, Jr., Lewert RM, Blas BL. Sero epidemiology of Schistosomiasis japonica by elisa enzyme linked immuno sorbent assay in the philippines 1. Underestimation by stool examination of the prevalence of infection in school children. Am J Trop Med Hyg. 1983;32(6):1322-34.

12. Zhou YB, Yang MX, Wang QZ, Zhao GM, Wei JG, Peng WX, et al. Field comparison of immunodiagnostic and parasitological techniques for the detection of Schistosomiasis japonica in the People's Republic of China. Am J Trop Med Hyg. 2007;76(6):1138-43.

13. Zhou YB, Yang MX, Tao P, Jiang QL, Zhao GM, Wei JG, et al. A longitudinal study of comparison of the Kato-Katz technique and indirect hemagglutination assay (IHA) for the detection of Schistosomiasis japonica in China, 2001-2006. Acta Trop. 2008;107(3):251-4.
