# Supplementary material for: Diagnostic test accuracy for detecting Schistosoma japonicum and S. mekongi in humans: A systematic review and meta-analysis
Source: PLoS Negl Trop Dis. 2021 Mar 17;15(3):e0009244. doi: 10.1371/journal.pntd.0009244 (PMC7968889; doi:10.1371/journal.pntd.0009244)
Supplement: S2 Table — (DOCX) [file pntd.0009244.s006.docx]

**S2 Table. Index test specific 2x2 data (TP, FP, FN, TN) of studies reported *S. mekongi*.**

| **SL** | **Author (reference)** | **Reference standard** | **Index test** | **TP** | **FP** | **FN** | **TN** |
| --- | --- | --- | --- | --- | --- | --- | --- |
| 1 | Nickel et al., 2015 [1] | Kato-katz (4 slides from 2 stools) | Combined ELISA, AWE, SEA of S. mansoni antigen | 173 | 31 | 10 | 20 |
| 2 | Sayasone et al., 2015 [2] | Kato-Katz (3 slides from 3 stools) | FECT | 3 | 23 | 0 | 459 |

*Note: AWE- adult worm antigen; ELISA-Enzyme-Linked immuno sorbent assay; FECT-Formol-ethyl acetate sedimentation concentration technique; SEA- Soluble egg raw antigen; TP-Ture positive; FP-False positive; FN-False negative; TN-True negative.*

**References**

1. Nickel B, Sayasone S, Vonghachack Y, Odermatt P, Marti H. Schistosoma mansoni antigen detects Schistosoma mekongi infection. Acta Trop. 2015;141:310-4.

2. Sayasone S, Utzinger J, Akkhavong K, Odermatt P. Repeated stool sampling and use of multiple techniques enhance the sensitivity of helminth diagnosis: a cross-sectional survey in southern Lao People's Democratic Republic. Acta Trop. 2015;141:315-21.
